# Multi-level modeling with nonlinear movement metrics to classify self-injurious behaviors in autism spectrum disorder

**DOI:** 10.1038/s41598-020-73155-4

**Published:** 2020-10-07

**Authors:** Kristine D. Cantin-Garside, Divya Srinivasan, Shyam Ranganathan, Susan W. White, Maury A. Nussbaum

**Affiliations:** 1 Department of Industrial and Systems Engineering, Virginia Tech, Blacksburg, VA USA; 2 Department of Statistics, Virginia Tech, Blacksburg, VA USA; 3 Center for Youth Development and Intervention, Department of Psychology, University of Alabama, Tuscaloosa, AL USA

**Keywords:** Engineering, Diagnostic markers

## Abstract

Self-injurious behavior (SIB) is among the most dangerous concerns in autism spectrum disorder (ASD), often requiring detailed and tedious management methods. Sensor-based behavioral monitoring could address the limitations of these methods, though the complex problem of classifying variable behavior should be addressed first. We aimed to address this need by developing a group-level model accounting for individual variability and potential nonlinear trends in SIB, as a secondary analysis of existing data. Ten participants with ASD and SIB engaged in free play while wearing accelerometers. Movement data were collected from > 200 episodes and 18 different types of SIB. Frequency domain and linear movement variability measures of acceleration signals were extracted to capture differences in behaviors, and metrics of nonlinear movement variability were used to quantify the complexity of SIB. The multi-level logistic regression model, comprising of 12 principal components, explained > 65% of the variance, and classified SIB with > 75% accuracy. Our findings imply that frequency-domain and movement variability metrics can effectively predict SIB. Our modeling approach yielded superior accuracy than commonly used classifiers (~ 75 vs. ~ 64% accuracy) and had superior performance compared to prior reports (~ 75 vs. ~ 69% accuracy) This work provides an approach to generating an accurate and interpretable group-level model for SIB identification, and further supports the feasibility of developing a real-time SIB monitoring system.

## Introduction

Autism spectrum disorder (ASD) is a pervasive neurodevelopmental disability marked by communicative, social, and behavioral impairments^1^. Self-injurious behavior (SIB), including head banging and self-hitting^2^, is reported in roughly half of people with ASD^3–5^. SIB is a leading cause of hospitalization among people with ASD and can lead to physical damage such as lacerations and contusions^2,6^. These behaviors can be repetitive or rhythmic, though behavior presentations vary widely^2^. Applied behavioral analysis thus suggests that caregivers perform a functional assessment (FA) to determine potential triggers of SIB^7–9^. To complete an FA, clinicians or trained caregivers observe and record details about events preceding, during, and following SIB^9^. Accuracy of an FA can suffer if caregivers are not adequately trained, though, and if events are recalled after they occurred^10,11^. Observations also can differ between caregivers and clinicians, and can be challenging to track consistently due to other stressors and contextual influences on behavior^8,10,12–14^. FAs require detailed observations and extensive note-taking on complex data, and these requirements lead to a time-consuming process^15^. Further, the behavior of interest may not occur during the observation period of an FA. The lack of SIB during an FA may be due to the time window of assessment or the absence of triggers in a specific environment^16^, which in turn may necessitate a repeated FA and add to the required completion time. Thus, traditional manual methods are often inefficient^10,14–16^, and as such do not support the widespread need for care across contexts.

An accurate SIB tracking system might overcome these challenges if the system could identify triggers and inform and evaluate management. Sensing technology has the potential to comprehensively, objectively, and accurately track movement for people with SIB, as supported by previous research on behavioral monitoring for non-SIB behaviors in ASD^17–19^. Nonwearable and wearable technologies, such as embedded camera systems or accelerometers in everyday items (e.g., cellphones), could record data continuously for SIB monitoring without requiring high levels of caregiver or clinician compliance^19,20^. Wearable accelerometers address limitations of nonwearable technology, such as restricted field of view and privacy concerns^21,22^, and were selected for the current study to reflect caregiver preferences from our previous work^23^. Caregivers in that work indicated a need for data collection methods applicable in school and at home, and they suggested that children with SIB would accept wearable technology if noninvasive, comfortable, and discrete attachment methods were possible. Accelerometers have also been shown to provide sufficient data to detect repetitive motions among individuals with ASD, with 80–97% accuracy using wrist and/or back sensors^24,25^, though use for SIB detection has not been previously explored.

In conjunction with wearable technology, SIB monitoring requires effective modeling. Earlier findings support the feasibility of tracking behaviors in ASD, specifically stereotypical motor movements (SMM) such as hand-flapping or rocking, which may relate to SIB and be similarly repetitive and rhythmic^2^. Machine learning classifiers applied to accelerometry data—including decision trees^26^, neural networks^27,28^, and support vector machines^7^—detected SMM with accuracies up to 99%. However, there is very limited extant evidence for SIB classification. Previous work on SIB detection, to our knowledge, is limited to two studies that either created classifiers from trained actors imitating aggressive behaviors^18^ or focused on SMM with one example that could be considered SIB^29^. The former study extended models that were trained on imitated movement to one child with SIB, and found that classification with individual accelerometry data yielded accuracies on the order of 60–70%^18^. Classifiers may have had stronger performance if trained on natural data, (versus simulated SIB), and their generalizability is unknown when used on more than one participant with more than one behavior. One study also examined aggression towards others among youth with ASD^30^. Naturally-collected episodes of aggression were classified with high accuracy using physiological and movement sensors (area under the curve: AUC = 71–80 for individuals; AUC = 69 for group performance), though SIB was not included in the activities of interest^30^. Importantly, sensory aversions prevalent in SIB^31^ may preclude the physiological sensors that require skin contact, which were used in Ozdenizci et al.^30^, so other sensor and classification methods may be preferable for our application.

Classification models in earlier studies were typically specific to each participant, with training and testing completed on each individual^18,29^. When group-level models were employed, accuracy levels tended to decrease, for example from 80% for individual models to 69% for group-level models in Ozdenizci et al.^30^. Additionally, machine-learning based classification methods used in earlier studies (e.g., SVMs or neural networks) can have low interpretability, and other more accessible models should also be explored, such as regression^30^. Interpretable models could provide information about predictors of SIB onset, which would be particularly relevant for clinicians and caregivers seeking to manage this behavior (see Cantin-Garside et al.^23^, Dunlap et al.^12^ and Williams et al.^9^ for further discussion on the need to capture triggers of SIB). Multilevel regression models with varying intercepts and slopes could account for the variability among individual diagnoses of ASD and in presentations of behavior^32,33^, though such a model has yet to be applied to SIB.

In our previous study, we examined featureless classification methods to detect the presence of SIB and classify the type of SIB among individuals and groups of individuals with ASD^34^. Using data from wearable accelerometers as input, accuracy was up to 99.1% for individual models, and up to 94.6% for models specific to SIB type. However, the detection models that incorporated all participant data had substantially poorer performance (accuracy = 48.8%), likely due to the inter- and intra-individual variability in SIB and activity levels. The current study is a secondary analysis of data described in Cantin-Garside et al.^34^, and employed a multi-level modeling technique that includes motor variability features to address this limitation^34^.

Including additional features, such as metrics from movement variability, may optimize system performance. In general, variability can be described using linear measures included in SMM classification^27^, such as the standard deviation, as well as using nonlinear dynamics measures, such as entropy^35^. Linear measures of variability consider variability in systems to stem fundamentally from noise, and utilize statistical dispersion measures such as the standard deviation to quantify the variability in time-domain signals^36^. Nonlinear measures of variability, such as entropic and fractal measures, can quantify the temporal evolution of movement^36^. Dynamical systems theory suggests that human movement changes and evolves over time, as governed by a deterministic process^37^. Dynamical systems analyses separate variability in a movement process into chaotic vs. deterministic variability components^36,38^. On the other hand, non-linear measures based in chaos theory and dynamical systems analysis consider the evolution of changes in a system over time.

Prior work classifying SMM in ASD has used time- and frequency-domain features^25^, or has focused on relatively simple measures of variability such as the standard deviation and variance, with the latter yielding frequent false positives^27^. Nonlinear movement variability features could improve classification model performance by capturing the underlying variability in SIB movements^39^, even if the SIB changes between episodes. Dynamical systems theory may be relevant to SIB, since nonlinear components were found in the temporal patterns of SIB, though differing within and between individuals^40^. More complex temporal patterns also emerged in the presence of SIB^40^, and recent work suggests that movements become increasingly complex as a child with ASD transitions to an episode of SIB^41^. This complexity can be captured by measuring nonlinear variability in the movements of individuals with ASD and SIB, as further explained below in the description of nonlinear motor variability metrics.

Prior work also has found that nonlinear measures, such as entropy, are indicative of diagnosis when applied to motor control in ASD. For example, children with ASD had decreased dynamical complexity during quiet stance compared to typically-developing children^42,43^, although people with stereotypy showed greater linear variability (standard deviation) during postural sway^43^. Given that they can distinguish between neurotypical and pathological movements, these methods could capture changes in pathology within an individual (i.e., detecting health changes such as early signs of aggression). Variability has also been associated with other pathological behaviors^35,36,44^ and the progression of health conditions^45,46^, and thus could reflect changing risk of SIB in ASD as well. To our knowledge, though, only one study employed a nonlinear approach (recurrence quantification analysis, described below) to classify motion in ASD, and found that the additional nonlinear features of movement variability improved classification accuracy by 5–9%^39^. Although the analysis in Großekathöfer et al.^39^ was performed on SMM, their results could generalize to SIB, which is similarly repetitive and rhythmic.

In summary, SIB is one of the most dangerous behaviors in ASD, and a monitoring system could address the limitations of traditional tracking methods. Predictive modeling with features capturing nonlinear motor variability has the potential to provide superior performance vs. more traditional methods used in related ASD research. However, sensor-based behavioral monitoring for SIB has not yet been explored. A long-term goal of our research is to develop a real-time SIB monitoring system that can collect continuous movement data, alert the caregiver before SIB onset, and assist in management methods (e.g., redirecting the individual with SIB towards a different task). To this end, we aimed in the present study to develop an interpretable and generalizable model to classify a variety of behaviors among a range of participants, specifically by:

1. Utilizing dynamical systems theory to extract measures of nonlinear motor variability as features in an SIB prediction model
2. Building a multilevel logistic regression model with variable intercepts and slopes to account for inter-individual variability

## Materials and methods

### Participants

Data used here were obtained in Cantin-Garside et al.^34^ and are briefly summarized below, with additional information in Supplementary Material. Children with SIB and ASD were recruited through the university-affiliated psychology clinic and through the authors’ networks. Caregivers were pre-screened to confirm inclusion criteria: (1) children aged 5–14 years, reflecting heightened aggression in childhood^47,48^; (2) diagnosis of ASD; (3) SIB episodes > 3/hour, to ensure multiple episodes during the 1–3 h sessions; (4) fluency in English; and (5) home within driving distance of the noted Center. Note that the last inclusion criterion required non-representative convenience sampling. All adult participants provided informed consent, and qualifying children (> 7 years of age and of developmental level) provided assent before any data collection. Caregivers provided informed consent for their children who were younger than 18 years of age. The Virginia Tech Institutional Review Board (IRB) reviewed and approved all experimental procedures. All experimental methods were completed in accordance with relevant guidelines and regulations.

Eleven participants (5–14 years, M = 9.5, SD = 3.0) and their caregivers completed the study. Sessions lasted 35–147 min, providing more than 1000 min of data and > 200 episodes of SIB. Ten of the 11 participants exhibited SIB (participants 1–4 and 6–11, denoted as “P#”) with 18 different types (Table 1). All participants wore the wrist sensor, and the limited sensor configurations of P1, P8, and P9 precluded the use of other sensors in the group-level model. To include all participants in one group-level model, only the wrist sensor was considered. In contrast, data from 2 to 6 tri-axial accelerometers (Table 1) were used in individual-level models.

**Table 1:** Participant identifier, type of SIB shown during the session, total duration (seconds), and sensors worn.

	Behavior(s)*	Total duration (s)	Sensors worn
P1	Repeated foot to surface (1)	13	Wrist, waist (part 1) Wrist, waist, pockets, ankle (part 2)
	Repeated hand to surface (2)	6	
	Head hitting –with object (3)	20	
P2	Finger picking (picking skin off of fingers) (4)	87	Wrist, waist, pockets, ankles
	Scratching (5)	28	
P3	Heel to surface (1)	66	Wrist, waist, pockets, ankles
	Hand to surface (2)	7	
P4	Self-biting (hands, arms) (9)	301	Wrist, waist, pockets, ankles
	Self-hitting (10)	80	
	Pulling teeth (11)	33	
	Eye-gouging (jabbing eye with hand) (12)	79	
	Jabbing pelvic region (13)	16	
	Jabbing throat – location of prior tracheotomy (14)	46	
	Hitting chin/jaw with heel of hand (15)	66	
P6	Foot to surface (1)	2	Wrist, waist, pockets, ankles
	Hand to surface (2)	15	
	Repeatedly pulling on teeth using string/object (17)	256	
	Blowing on fingertips (16)	322	
	Spinning (18)	155	
	Flapping (19)	14	
	Jumping/flapping arms (20)	25	
	Jump/spin (21)	6	
P7	Finger picking (4)	322	Wrist, waist, pockets, ankles
P8	Foot to object (1)	2	Wrists, pockets
	Hand to surface (2)	4	
	Throwing body against object or surface (6)	22	
P9	Finger picking (4)	229	Wrist, waist, ankles
	Lip picking (picking skin off of lip) (7)	13	
	Head to wall (8)	14	
P10	Hands to surface (2)	9	Wrist, waist, pockets, ankles
	Finger Picking (4)	9	
	Scratching (5)	2	
	Head to wall (8)	20	
	Self-biting (9)	39	
	Self-hitting (10)	4	
	Eye-gauging (12)	58	
	Pulling ear (22)	209	
	Flapping (19)	5	
P11	Finger picking (4)	97	Wrist, waist, pockets, ankles
	Hair pulling (23)	73	

The wrist sensor was commonly worn among all participants.

### Study overview

After obtaining consent, the lead author, or the caregiver guided by the author, secured sensors on the child where tolerated. Demographic information was obtained, including potential SIB triggers identified by the caregiver. A trained clinical psychology doctoral candidate confirmed ASD diagnosis using standard tools (i.e., ADOS-2)^49^. The examiner was research reliable in administration and scoring of the ADOS-2, and was supervised by a licensed clinical psychologist who was also reliable (SWW). Subsequently, movement sensors (see “Instrumentation”), video cameras, and 2–3 observing researchers monitored each child during free-play. Researchers instructed the caregivers to respond to SIB as if at home. If sufficient SIB episodes failed to occur during free play, caregivers had the option to prompt SIB in a controlled fashion with a commonly-used procedure (Standardized Observation Analogue Procedure, SOAP)^50^. The session ended when either: (a) > 3 episodes of SIB were observed, or (b) participants or researchers stopped the session to prevent escalating behavior. At the end of the session, participants were compensated for their travel and time, and the children were presented with a selected toy.

### Instrumentation

Tri-axial accelerometers (ActiGraph GT9X Link, www.actigraphcorp.com) were used to track participant movement (sampling frequency = 60 Hz) throughout the session. Earlier work found that these particular sensors were both reliable and accurate when used with children and adolescents^51^ for tracking movement among both pathological and healthy populations^52^. A maximum of six sensors were placed on/in the wrists, waist, pockets, and ankles as accepted by the participant. Sensor choice and placement reflected prior research that found high reliability and high accuracy when classifying activities with movement sensors on either the wrist or torso^25,52,53^. Ankle sensors were also included as potentially necessary to capture lower-body injurious behaviors^18^. Three Go-Pro cameras and an overhead camera recorded videos for each child as “ground truth”.

### Data processing and analysis

**Figure 1:**
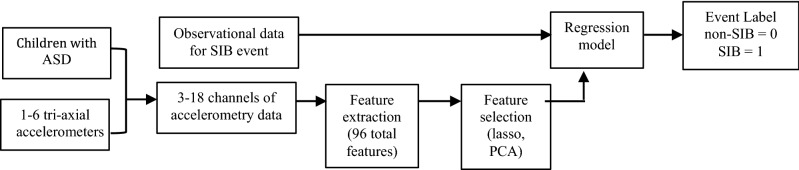
Overview of the data analysis and modeling process.

Sensor data were exported into MatLab (R2018a, MathWorks), which was used for data analysis and modeling (using an Intel, dual-core, 2.9 GHz CPU). Accelerometer data were labeled as non-SIB events (0) or SIB (1) using the ground truth video data and annotations from in-session observations. Before the session began, members of our research team discussed the SIB that parents described during pre-screening. Behaviors were defined by watching the children individually and captured by terms provided by their caregivers (e.g., eye-gouging). Members of our team also discussed behaviors observed in sessions, both during and after the session, for consensus building. Behavioral definitions were further clarified prior to data labeling. Multiple researchers annotated and discussed the video data before labeling raw accelerometry files (see Fig. 1 for an overview of the modeling process, and Cantin-Garside et al.^34^ for details on consensus-building for SIB labels)^34^.

Raw sensor data were filtered using a 4th order, low-pass, recursive Butterworth filter, with a cutoff frequency of 20 Hz. Filtered data were used to obtain time- and frequency-domain features. Based on prior work^54,55^, raw data were used for extracting features of nonlinear motor variability (see “Derivation of nonlinear metrics of motor variability”). For continuous analysis of discrete data, all data were segmented into 2-s sliding windows with a 1-s overlap^7,27^. This short time window was used to minimize delays, which was considered important for real-time monitoring and reflects the potential for relatively short “bursts” of SIB.

### Feature extraction

Three sets of features were extracted: (1) features in the time-domain; (2) features in the frequency-domain; and (3) nonlinear metrics based on chaos theory and dynamical systems. As discussed above, the use of time- and frequency-domain features is supported by prior findings on classifying SMM^7,18,22,27^, with nonlinear motor variability features included to capture the dynamical complexity of motion and to improve classifier performance^56,57^. Table 2 lists the features extracted for each channel. The presence (1) or absence (0) of a prompt to instigate SIB (from SOAP) was also included initially during feature selection (see “Feature selection”); all caregivers except for Participant 4 opted to use SOAP at least one time during their session.

**Table 2:** Time, frequency, and nonlinear motor variability features.

Feature type	Time domain	Frequency domain	Nonlinear motor variability
Number of features	19 features × 3 channels = 57 features	4 features × 3 channels = 12 features	9 features × 3 channels = 27 features
Features	Channel cross-correlation coefficient Mean difference between channels Variance Local Minima Count Local Maxima Count Peak Minimum Percentiles from amplitude probability distribution: 1, 10, 25, 50, 75, 90, 99 Zero Crossings Average Root mean square (RMS) Jerk	First two frequencies of FFT First two corresponding amplitudes	Detrended fluctuation analysis (DFA): Exponent (α) Entropy: Sample entropy Cross sample entropy Recurrence Quantification Analysis Metrics (RQA): Recurrence Determinism Laminarity Divergence Maximum diagonal length Trapping time

#### Derivation of nonlinear metrics of motor variability

Nonlinear metrics of motor variability were extracted by first reconstructing the phase space of the raw sensor data^36,58^. Phase space represents the states (“state space”) of dynamical system behavior in a plot, and this reconstruction involves creating *M* copies of the original time series $x$, where *M* is the embedding dimension, using a time delay $(\tau )$^36,59^. Time delay was determined using two methods: (1) the first minimum of the average mutual information function^60,61^; (2) the delay when the time series autocorrelation was less than $e$^-1^^56,62^. Time delay was determined using both methods separately for SIB and non-SIB events (see^36,56,59^ for additional details). The selected $\tau$ was the value for which both methods converged, and was similar for both SIB and non-SIB ($\tau =5)$. The resulting embedding dimension (*M*) was 4, derived from the global false nearest neighbor analysis method^61,63^. Both $\tau$ and *M* values here are similar to prior work on the nonlinear variability of human motion^61^. State space was reconstructed as embedding vectors $X\left(t\right)$ in the form:

1 $$X\left(t\right)=\left[x\left(t\right),x\left(t+\tau \right),x\left(t+2\tau \right),\dots , x\left(t+\left(M-1\right)\tau \right)\right]$$

such that $t=1,\dots ,N-\left(M-1\right)$. Delay reconstruction was used to create the phase space, which produced consistent results in other work^61^. The parameters described below were then calculated using the reconstructed phase space.

#### Entropy

Sample entropy (SaEn) was used to quantify the complexity of the acceleration signals, with low values indicating low complexity^56,64,65^. SaEn was calculated using the following steps:

1. Compute ${C}_{t}^{M}\left(r\right)=\left\{number of X\left(i\right) such that |\left|X\left(t\right)-X\left(i\right)\right|{|}_{\infty }\ll r\right\},$where *C* is the probability that the vector *X(i)* is within the tolerance threshold, $r=0.2*STDDEV\left(x\right)$ of *X(t)*, *M* is the embedding dimension, and $t\ne i$
2. Find ${\phi }^{M}\left(r\right)=\sum_{t=1}^{N-M+1}\frac{{C}_{t}^{M} (r)}{N-M+1},$ which is the average of ${C}_{t}^{M} \left(r\right)$where *N* is the number of data points from the signal.
3. Calculate $$SaEn=-ln\frac{{\phi }^{M+1}\left(r\right)}{{\phi }^{M}\left(r\right)}$$

#### Cross-sample entropy

Cross-sample entropy has not been explored in models classifying ASD motion, though was determined here between each sensor channel and used in our feature set. Cross-sample entropy parallels SaEn in its estimation, but examines the difference between one data stream and another data stream^56,66^. Lower values imply similarity and synchronicity between the two data streams^64^.

#### Recurrence quantification analysis

Recurrence quantification analysis (RQA) was performed with a MatLab toolbox^67,68^ to evaluate phase space predictability and intermittency^56,69^. An RQA map is first constructed through a distance matrix comparison. A distance matrix $(\mathrm{DM})$ consists of elements (${\mathrm{DM}}_{ij}$) that are Euclidian distances (${\mathrm{DM}}_{ij}=d[X\left(i\right),X\left(j\right)]$) between embedding vectors $X\left(i\right)$ and $X\left(j\right)$^56^. ${\mathrm{DM}}_{ij}$ elements are then compared against a threshold determined by recurring dynamical trajectories, with elements = 1 for ${\mathrm{DM}}_{ij}$< threshold, indicating recurrent points returned to a previous location, and = 0 otherwise^39,56^. The selected threshold guarantees that the percentage of recurrent points remains within 0.1–2% of the total recurrent elements^56^. RQA can be evaluated using several measures^56,69,70^, with the following selected as reliable for human subject research^56,66,69–71^:

1. Recurrence—regularity of the time series as the percentage of recurrent points
2. Determinism—percentage of consecutive, diagonally-aligned recurrent points indicating signal periodicity and predictability; this relates to the inverse of the largest positive Lyapunov exponent, because longer diagonals imply deterministic versus chaotic movements
3. Laminarity—percentage of vertically aligned recurrent points, indicating signal stability (similar to determinism)
4. Divergence—inverse of the maximum diagonal line segment, related to the maximal Lyapunov exponent
5. Maximum diagonal length—proportional to the inverse of the maximal Lyapunov exponent, indicating the longest duration of periodicity
6. Trapping time—mean vertical line length, indicating the duration of the trapped state, reflecting signal constancy

#### Detrended fluctuation analysis

Detrended fluctuation analysis (DFA) was used to quantify the persistence of SIB movements. DFA exponents (α) were calculated for every time segment to assess long-range correlations^36,72^, with persistence indicated by α > 0.5 for time series deviations that continue in the same direction, and anti-persistence by α < 0.5 for deviations that continue in the opposite direction^72^. DFA has been used in analyses of motor control for ASD, with evidence of long-range correlations (persistence) during a drawing task^73^.^.^DFA has also been applied to capture the predictability of a movement, specifically when walking^72,74^. Persistence typically degrades in pathology. The underlying long-range correlations are altered in disordered movement, compared to consistent correlations in healthy movement (Goldberger et al.^75^). This finding remains evident irrespective of whether the outward appearance of pathological behavior appears more restricted or more chaotic^75^.

### Feature selection

The least absolute shrinkage and selection operator (lasso) method was used to address multicollinearity, to remove redundant features, and to determine the sparsest model when considering all 96 features and all sensors^76^. This method directly selects variables that most contribute to the model. Principal component analysis (PCA) was then used for dimensional reduction, by finding the optimal combination of the 59 selected variables (see Supplementary Materials, Table S2 for further information on variable selection and loadings)^77^. This feature selection approach is capable of characterizing data despite high variability between participants. PCA output was subsequently used as input to a multilevel logistic regression model (MLR).

### Regression modeling

A multilevel logistic regression (MLR) model was created with variable slopes and intercepts. The latter were used to account for high inter-subject variability^32^, which could be particularly relevant for ASD. Further, this model relied upon data from other individuals when classifying an episode of SIB to improve the overall performance of the entire group. This aspect of the model is particularly relevant, as SIB episodes can be sparse, depending on the individual and type of SIB. Specifically, the model can be written as:

2 $${Y}_{i}=logi{t}^{-1}\left({\alpha }_{j\left[i\right]}+{\sum }_{k}{\beta }_{j\left[i\right],k}{X}_{k}\right), i=\mathrm{0,1},\dots I;j=\mathrm{1,2},\dots ,J;k=\mathrm{1,2},\dots ,K$$

where *i* indexes over events, *j[i]* is the index of a subject who exhibits event *i*, *k* indexes over the features $X$, and ${Y}_{i}=1$ is the outcome variable if the event is SIB vs. 0 otherwise. Intercepts ${\alpha }_{j\left[i\right]}$ and feature slopes ${\beta }_{j\left[i\right], k}$ are variable for each subject, and both can be modeled linearly as:

3 $${\alpha }_{j}\sim N\left({U}_{j}{\gamma }_{j}^{\alpha }, {\sigma }_{\alpha }^{2}\right); {\beta }_{j,k}\sim N\left({V}_{j,k}{\gamma }_{j,k}^{\beta }, {\sigma }_{\beta k }^{2}\right)$$

where ${U}_{j}$ and ${V}_{j,k}$ are potential features, with corresponding linear coefficients ${\gamma }_{j}^{^{\prime}}$ specific to the individual level, and modeling error variances ${\sigma }^{2}$.

### Evaluation

Data were balanced and randomly selected following a 8:2 training/validation:testing ratio, so as to build a robust model and to test the built model^78^. SIB events are relatively rare compared to non-SIB events, which leads to a skewed distribution as found in prior work with ASD-related behaviors^7,39,79^. SIBs here lasted for about two seconds at minimum, though more subtle movements, such as picking, lasted longer, which lasted ~ 10 to 90 s. SIB and non-SIB data were balanced as in other work to address skewness^28,39,79–81^. Balanced data were used for training, and tenfold cross-validation was used^18,26,30^. This validation method was implemented to reflect the likely use cases in SIB interventions, including training and validating a model using data from each unique individual. SIB management is highly specific to the individual and the demonstrated behaviors, thus requiring representative data. Movement classification for SIB must therefore reflect the need for highly customizable tracking methods and account for heterogeneity. Two datasets were used for testing model generalization: (1) balanced data; and (2) natural, unbalanced data reflecting the ratio of SIB:non-SIB in the complete dataset. These testing methods were used to examine the potential use of a model pre-trained on controlled data for application to natural, unbalanced datasets. All data were randomly selected from across the duration of a given session, and observations were assumed to reflect the entire dataset^81^.

Outcome measures were calculated for each model (MLR, and the models described below), with classification performance (accuracy, specificity, precision, recall, and F-score) calculated for each classification method^82^. Training and testing time were also computed for all developed models to assess the potential for application to real-time monitoring.

### Model comparisons

Additional group-level models were trained, validated, and tested, for the purpose of comparison with the MLR model (“MLR – variable intercepts and slopes”). These additional models were of five different types:

1. Logistic regression (LR) with variable intercept only (“LR—variable intercept”). This was used to compare the MLR with a less complex model, while still accounting for participant variability.
2. LR without variable slopes or intercepts (“LR—no variable terms”). This model was included to compare the MLR with a model that does not consider participant-level variation.
3. Two-way interaction model (stepwise LR), with included terms determined by BIC (“LR—stepwise”). This model was included to compare the MLR with higher-order, nonlinear models, and to evaluate the effect on accuracy when including terms with lower interpretability but potentially higher predictive power.
4. Participant-level LR models (“LR-ind”), one for each of the 10 who exhibited SIB. These models were included to compare the group-level MLR with highly specific modeling that may have low generalizability, yet high accuracy.
5. Several models using machine learning methods: k-nearest neighbors (“kNN”), with k = 11 selected through optimization; support vector machines (“SVM”), and decision trees (“DT”). These three models were included to compare the MLR with previously-employed methods demonstrating strong individual (though not group-level) performance in other ASD applications and our previous featureless work (Cantin-Garside et al.^34^), and to compare the MLR with “black-box” models with lower interpretability but typically high predictive power.

## Results

### Dimensional reduction

Fifty-nine variables were selected from lasso and were then input in PCA for the group-level MLR model; these variables included both linear and nonlinear features of motor variability features from each sensor channel. Lasso results, though, excluded the prompt variable. Means and standard deviations of select nonlinear motor variability features are provided in Supplementary Materials, Table S1. PCA generated 12 principal components (PCs). Coefficients and the explained variance of each PC are provided in Supplementary Materials, Table S2, and a summary of PCs and top loading variables is provided below in Table 3. PC1 explained 23% of the variance, with loadings primarily from frequency-based measures and measures capturing sudden or sharp movements (e.g., jerk, peak). Measures of the Z channel (vertical) loaded primarily on PC2, with coefficients > 0.3 for mean absolute value and RMS. Nonlinear motor variability metrics had coefficients up to ~ 0.6 in some PCs. Nonlinear metrics from RQA had coefficients > 0.4 on PC6 (Z channel), > 0.3 on PC8 (X channel), and > 0.4 on PC9 (Y channel), while SaEn had coefficients > 0.3 on PC10 and cross-sample entropy (ZY) had coefficients > 0.3 on PC12. The components listed above with nonlinear variable loadings > 0.3 accounted for 11.1% of the total variance (3.6, 3.0, 2.6, 2.5, and 1.9% respectively). All 12 components contributed to 65.6% of the group data variance.

**Table 3:** Summary of each PC, with top-loading features and total explained variance per PC.

Principal components											
PC1	PC2	PC3	PC4	PC5	PC6	PC7	PC8	PC9	PC10	PC11	PC12
**Feature**											
Second FFT amplitude X	RMS Z	Mean absolute value of X	Mean absolute value of Y	50th percentile Y	Trapping time Z	90th percentile Z	Recurrence X	Recurrence Y	Sample entropy X	25th percentile X	Corr. coefficient YZ
Jerk X	Mean absolute value of Z	Minimum X	25th percentile X	50th percentile Z	Recurrence Z	50th percentile Z	Maximum diagonal length X	Trapping time Y	Sample entropy Z	10th percentile X	Cross- sample entropy YZ
Jerk Y	Minimum Z	99th percentile X	10th percentile X	90th percentile Z	Laminarity Z	50th percentile Y	Trapping time X	Maximal diagonal length Y	Sample entropy Y	Mean absolute value of Y	Corr. coefficient XY
Jerk Z	Peak Z	Mean absolute value Z	Mean absolute value Z	1st percentile Y	Local minima count X	1st percentile Z	Determinism X	50th percentile Z	Second FFT Peak Z	99th percentile X	Maximum diagonal length Z
First FFT amplitude X	Determinism Z	25th percentile X	RMS Z	99th percentile Y	First FFT Peak Z	99th percentile Z	First FFT peak Y	Divergence Y	Second FFT peak Y	Mean absolute value of X	First FFT peak Z
**Explained variance**											
**23.011**	**8.014**	**6.323**	**5.270**	**3.900**	**3.562**	**3.249**	**3.006**	**2.624**	**2.455**	**2.269**	**1.876**

Bold values indicate significant features in the model (*p* < 0.05).

Table 4 summarizes results using the MLR. PC1 and PC12 were both significant features when considered across participants, though not when varying with participant level. The intercept, along with PCs 2, 5, 6, 7 and 9, were only significant features when considering participant levels, and not when fixed. PCs 3, 8, 10, and 11 were significant features both when fixed and when randomly varying with participant level. PC4 was not significant in the model, either when fixed or when varying with participant level.

**Table 4:** Multi-level logistic regression parameter values for the group-level model including all 10 participants.

Parameters	Fixed effect	Varying with participant (Parameter|Par)
Intercept	− 0.363	**1.192**
PC1	**0.033**	1.456e−15
PC2	− 0.021	**0.217**
PC3	**0.247**	**0.274**
PC4	− 0.001	1.712e−08
PC5	− 0.031	**0.228**
PC6	0.060	**0.146**
PC7	0.003	**0.255**
PC8	**0.239**	**0.084**
PC9	0.028	**0.181**
PC10	− **0.273**	**0.185**
PC11	**0.333**	**0.250**
PC12	**0.132**	2.112e−09

Bold values indicate significant features in the model (*p* < 0.05).

### Classifier performance

Tables 5 and 6 respectively summarize results regarding training time, accuracy, specificity, precision, recall, F-scores, and adjusted R^2^ for validation and testing of group-level models. Training times for MLR, stepwise LR, and cubic SVM were 2–4 times longer than for other classifiers (10^–2^ vs. 10^–6^ s/observation), though this same difference was not reflected in testing times (all times within 10^–6^–10^–5^ s/prediction). MLR had high accuracy (74.7%) and F-score (0.752) in validation, which decreased minimally when testing with balanced data (73.2% and 0.733). Accuracy and F-score decreased with unbalanced test data for MLR (69.1% and 0.184). Specificity, precision, and recall were all highest for MLR in validation (~ 0.73 to 0.77) and testing (~ 0.73 for all three measures). Adjusted R^2^ was the highest for MLR (0.502) and the lowest for LR without variable intercepts/slopes (0.106). When considering participant levels with only a variable intercept versus both variable intercept and slopes, most performance metrics decreased by ~ 2 to 5%. LR without variable intercepts/slopes had the lowest accuracy (64.0%) in validation, dropping to 47.0 and 56.1% for balanced and unbalanced data, respectively. Linear SVM had the lowest specificity and precision (0.599 and 0.631), while LR without variable intercept/slopes had the lowest recall (0.663), though this trend did not extend to testing results. Stepwise LR had the lowest specificity for both balanced and unbalanced test data (0.526 and 0.552, respectively). LR without variable intercepts/slopes had the lowest precision, recall, and F-score for balanced test data (0.455, 0.304, and 0.365, respectively). The kNN classifier had the lowest precision, recall, and F-score for unbalanced test data (0.064, 0.591, and 0.116, respectively), while LR without variable intercept/slopes had the lowest F-score for validation (0.648) and balanced test data (0.365).

**Table 5:** Validation results for group-level classifiers.

Classifier	Training time (s/observation)	Accuracy	Specificity	Precision	Recall	F-score	R^2^ adjusted
MLR—variable intercept and slopes	1.49E−02	0.747	0.728	0.738	0.766	0.752	0.502
LR—variable intercept	4.71E−04	0.705	0.676	0.694	0.734	0.713	0.332
LR—no variable terms	8.34E−06	0.640	0.617	0.634	0.663	0.648	0.106
LR—stepwise	8.49E−02	0.671	0.673	0.672	0.669	0.670	0.147
kNN, k = 11	1.49E−05	0.676	0.621	0.659	0.731	0.693	–
SVM—linear	1.66E−04	0.642	0.599	0.631	0.685	0.657	–
SVM—cubic	6.78E−03	0.696	0.657	0.682	0.734	0.707	–
SVM—Gaussian	1.28E−04	0.690	0.661	0.679	0.719	0.699	–
DT	9.94E−06	0.683	0.652	0.672	0.713	0.692	–

**Table 6:** Test results at the group level.

Algorithm	Test type	Prediction time (s/observation)	Accuracy	Specificity	Precision	Recall	F-score
MLR—variable intercept and slopes	1	5.98E−05	0.732	0.729	0.731	0.735	0.733
	2	5.70E−05	0.691	0.687	0.105	0.773	0.184
LR—variable intercept	1	1.54E−05	0.705	0.696	0.702	0.715	0.708
	2	1.66E−05	0.647	0.641	0.092	0.773	0.165
LR—no variable terms	1	1.33E−05	0.470	0.637	0.455	0.304	0.365
	2	1.07E−05	0.561	0.552	0.073	0.750	0.134
LR—stepwise	1	1.45E−05	0.488	0.526	0.487	0.450	0.467
	2	1.45E−05	0.561	0.552	0.073	0.750	0.134
kNN, k = 11	1	2.79E−05	0.643	0.567	0.624	0.719	0.668
	2	2.00E−05	0.591	0.591	0.064	0.591	0.116
SVM—linear	1	5.11E−05	0.619	0.612	0.617	0.626	0.622
	2	4.60E−05	0.551	0.542	0.072	0.750	0.131
SVM—cubic	1	4.76E−05	0.677	0.639	0.664	0.715	0.688
	2	4.67E−05	0.625	0.622	0.081	0.705	0.145
SVM—Gaussian	1	4.68E−05	0.671	0.641	0.662	0.702	0.681
	2	4.09E−05	0.645	0.646	0.076	0.614	0.135
DT	1	1.14E−05	0.695	0.676	0.688	0.715	0.701
	2	9.86E−06	0.640	0.638	0.082	0.682	0.146

Tables 7 and 8 show the classifier performance for validating and testing individual models, respectively. Time was on the order of 10^–5^–10^–3^ s/observation for training and 10^–4^–10^–3^ s/prediction for testing. Validation accuracy was higher overall (70.7–97.9%) when compared to group-level models (64.0–74.7%). Testing accuracy ranged widely, from 50.0–83.3% for balanced data to 27.2–95.7% for unbalanced data. Note that the unbalanced dataset, relative to the balanced dataset, did not lead to a substantial decrement in accuracy from validation to test set because there are substantially more behaviors labeled as non-SIB than SIB, and hence the label of non-SIB is easier to predict. Specificity ranged from 0.648–1 for validation datasets, and from 0.500–1 for balanced and unbalanced test data. Precision was between 0.692–1 for validation data, 0–0.818 for balanced test data, and 0–0.5 for unbalanced test data. The ranges of recall values were 0.746–1 for validation data and 0–1 for both balanced and unbalanced test data. F-scores ranged from 0.718–0.979 for validation data, 0–0.857 for balanced test data, and 0–0.667 for unbalanced data.

**Table 7:** Validation results for individual participants.

P	Training time (s/observation)	Validation accuracy	Specificity	Precision	Recall	F-score	R^2^ adjusted
1a	2.30E−03	0.893	0.857	0.867	0.929	0.897	0.850
1b	1.86E−03	0.979	1.000	1.000	0.958	0.979	1.000
2	1.61E−03	0.883	0.878	0.879	0.888	0.883	0.819
3	1.11E−03	0.803	0.770	0.785	0.836	0.810	0.548
4	7.84E−05	0.707	0.668	0.692	0.746	0.718	0.217
6	2.61E−04	0.857	0.838	0.844	0.876	0.860	0.605
7	1.55E−04	0.760	0.648	0.713	0.872	0.784	0.379
8	2.19E−03	0.935	0.870	0.885	1.000	0.939	1.000
9	1.74E−04	0.941	0.929	0.931	0.953	0.941	0.839
10	1.25E−04	0.772	0.797	0.786	0.747	0.766	0.365
11	2.24E−04	0.933	0.922	0.924	0.943	0.933	0.841

Note that 1a = first part of P1 session with only upper body sensors, and 1b = second part of P1 session with additional lower body sensors.

**Table 8:** Test results for individual participants.

P	Test type	Prediction time (s/prediction)	Accuracy	Specificity	Precision	Recall	F-score
1a	1	4.56E−03	0.833	0.667	0.750	1	0.857
1a	2	3.89E−03	0.833	0.800	0.500	1	0.667
1b	1	1.62E−03	0.500	1	0	0	0
1b	2	1.75E−03	0.917	1	0	0	0
2	1	2.94E−04	0.500	1	0	0	0
2	2	6.23E−04	0.938	1	0	0	0
3	1	1.80E−03	0.733	0.800	0.769	0.667	0.714
3	2	5.28E−04	0.533	0.517	0.067	1	0.125
4	1	1.04E−04	0.687	0.613	0.663	0.761	0.708
4	2	1.08E−04	0.577	0.525	0.263	0.833	0.400
6	1	1.66E−04	0.828	0.810	0.817	0.845	0.831
6	2	1.72E−04	0.543	0.514	0.117	1	0.209
7	1	2.97E−04	0.779	0.706	0.744	0.853	0.795
7	2	2.05E−04	0.272	0.238	0.057	1	0.108
8	1	2.30E−03	0.500	0.500	0.500	0.500	0.500
8	2	2.43E−03	0.538	0.500	0.143	1	0.250
9	1	2.76E−04	0.500	1	0	0	0
9	2	1.86E−04	0.925	1	0	0	0
10	1	1.76E−04	0.767	0.787	0.778	0.747	0.762
10	2	1.56E−04	0.673	0.655	0.140	1	0.246
11	1	3.67E−04	0.800	0.829	0.818	0.771	0.794
11	2	4.60E−04	0.957	1	0	0	0

Note that 1a = first part of P1 session with only upper body sensors, and 1b = second part of P1 session with additional lower body sensors.

## Discussion

We developed an interpretable model to identify diverse types of SIB among a range of participants. Traditional time- and frequency-domain features were used, along with features capturing nonlinear motor variability, as input to a multi-level logistic regression model capable of detecting SIB at the group level, with selected components from dimension reduction explaining > 65% of the data variance. The lasso method did not select the prompt variable for this group-level model (recall that this prompt represented the presence or absence of caregiver actions that were targeted at instigating SIB), indicating that this model explains the presence of SIB beyond an identified SIB trigger. This finding is consistent with a prior report^40^ that temporal patterns in SIB occur independent of behavioral or environmental influences (or “triggers”). Nonlinear motor variability features (e.g., from RQA and entropy) loaded on PCs that accounted for > 10% of the explained variance in the dataset. DFA features had moderate loadings on PCs, and these features were a novel addition to modeling for ASD.

Descriptive statistics of metrics of nonlinear motor variability from pooled SIB versus non-SIB events across participants showed little difference between the behavioral classes (Supplementary Materials, Table S1), which may explain the poor performance of a general group-level model without participant levels. However, upon examining one of the most severe behaviors (head banging) in one participant, nonlinear motor variability of SIB differed from non-SIB events (Supplementary Materials, Table S3). For example, DFA exponents for both non-SIB and SIB events in the Y and Z axes were slightly anti-persistent (< 0.5), indicating changes evolving in different directions over time. Though exponents remained < 0.5, there was a slight increase in DFA exponents for the Y and Z axes for SIB events compared to non-SIB, indicating more persistence in SIB events. Differences among other nonlinear metrics were evident for head banging in P9. Recurrence rate, for example, decreased for head banging in this individual compared to non-SIB events, indicating less regularity in SIB data. This finding opposes the common perception that repetitive behaviors are “regular”. There was a slight decrease in sample entropy for SIB in the Y and Z axes compared to non-SIB events, suggesting lower levels of complexity; however, cross-sample entropy increased during SIB, indicating higher levels of complexity between two channels of data. These findings may indicate that SIB occurs due to over/understimulation to seek system stability^83^ (“less” or “more” complexity)^84^ (see Mazefsky et al.^83^ for a review of emotional regulation in ASD, and Stergiou and Decker^84^ for a review of nonlinear dynamics and pathology).

Together, these results suggest that underlying nonlinear trends exist in the movements occurring during SIB. However, classic time- and frequency-domain features had the highest loadings on the first PC. These loadings indicate that jerk and FFT peaks are the strongest features of SIB, although nonlinear trends appear to differ between SIB and non-SIB. Consistent outcomes were found in prior work that used time- and frequency-domain features to accurately classify SMM^7,18,22,27^, and suggest that such features should be the first ones considered when creating a model to predict SIB. Further considerations for SIB modeling include variable intercepts and slopes. LR without variable intercepts or slopes performed inferiorly to LR with a variable intercept and inferiorly to MLR with both variable intercept and slopes. MLR with both variable intercept and slopes performed superior to all other classifiers, including commonly used machine learning algorithms implemented in other work^7,26^. These results imply that inter-individual variability also contributed to dataset variance.

This study is the first, to our knowledge, to incorporate nonlinear variability in addition to traditional time–frequency metrics to explain the variance in SIB movement data. Our findings suggest that movements in SIB can be described as a dynamical system with long-term deviations, which is consistent with prior evidence that stereotypical motor movements in ASD can be accurately detected using nonlinear features from RQA^39^. Similarly, our feature extraction revealed higher loadings for RQA features when compared to other nonlinear factors, and the associated principal components were significant in our MLR model. Our findings, along with those of^39^, indicate that RQA metrics could be critical in detecting repetitive and rhythmic motor movements (such as stereotypical motor movements, and SIB) in ASD.

Further, PCs with nonlinear variable loadings were significant in the MLR model. PC6 and PC9, with loadings primarily from RQA metrics from the Z and Y axes, respectively, were significant in the model only when randomly varying with participant level; these metrics were not sufficient to classify SIB unless including variable slopes, which indicates that nonlinear movement aspects are specific to each individual. Time- and frequency-domain features that loaded on PC1 (frequency components, jerk, and peak/minimum) were significant features in MLR when independent from participant levels, indicating that these variables could be predictive of SIB without considering individual variability; the only nonlinear metric of motor variability to which this finding applied was PC12 (cross-sample entropy). Other PCs with loadings from metrics of nonlinear motor variability (RQA for X on PC8, sample entropy on PC10) were significant only when considered as either a fixed or a variable effect, implying that features such as sample entropy vary consistently between participants while still explaining individual-level variability. Nonlinear measures were significant features of SIB when they varied with participants, which supports prior evidence that nonlinear components of movement are specific to individuals^85^.

We believe the current group-level model is the first to achieve accuracy of ~ 75% when identifying SIB among a diverse group of behaviors and participants. Previous research on classifying other repetitive motor movements^7,22,24–29,39^ and aggression^18,30^ has evaluated specific models trained and/or tested only on individual participants, and performance dropped from 80% with individual models to 69% when applied to the group of participants^30^. Though a similar decrease was also evident here, percent accuracy was ~ 6% higher than earlier group-level results. The increased performance we found may be due, at least in part, to the use of feature selection and dimensional reduction methods, along with the multi-level properties of our model that accounted for inter-individual variability. Also, a larger sample of participants was included here, compared to earlier modeling reports of ASD behaviors, with samples ranging from one to six^7,17,22,25–27,29^. Further, our participant pool encompassed 18 different behaviors across children 5–14 years of age, suggesting potential generality to a wider sample of children with ASD and SIB. Note that the 18 types of SIB mentioned in the study are a reflection of our study cohort and were an attempt to subtype SIB. We were not seeking to identify underlying subclasses of SIB, but instead to classify behaviors (as demonstrated naturally by our study cohort) automatically using technology (sensors and machine learning), rather than manually with traditional observation methods.

As in the work of^30^, regression showed promising results here compared to other classifiers; however, these earlier authors focused on aggression towards others, whereas our study applied regression on SIB data. MLR here had higher accuracy, specificity, precision, and recall compared to several commonly-used machine learning algorithms. These machine learning algorithms also detected SIB with high accuracy in our previous study using featureless data, though accuracy greatly decreased at the group-level (see Cantin-Garside et al. for further details)^34^. Multi-level regression with both variable slopes and intercept may be preferred for group data with variable behaviors that could be specific to an individual, and it may also be more accessible to interpretation than other machine learning algorithms. MLR had classifier performance superior to LR without varying intercept/slope, further emphasizing the potential importance of individualizing models for participants with ASD and SIB. MLR, though, was only inferior to several highly-specific participant models, which may not generalize beyond the participant.

The widely varying performance metrics across participants, however, could account for the unexplained variance in MLR. Several participants had either near perfect detection (e.g., P1) or quite poor detection (P8 or P9) in testing. This wide range could have resulted from the inconsistent amount of SIB data included (P9 had the shortest session of all participants), the different types of SIB, or the variable sensor configurations between participants. Of note, MLR only included the one sensor worn in common by all participants: the wrist sensor. This single wrist sensor may not be sufficient for all SIB types, such as head banging or kicking, and therefore might have led to decreased performance measures in the group model. Yet, despite having only one sensor to incorporate in the group model, MLR still showed superior performance to all other tested classifiers. Group-level classifiers may be more practical (i.e., efficient and generalizable) to implement in real-world applications, and the current results are promising for automatically identifying diverse SIB with minimally-invasive technology.

### Limitations and future work

Outcomes here provide initial groundwork toward creating a group-level classifier for SIB classification, yet further research is needed in several respects. Such work should include expanded data to improve classifier performance and to extend the model for more individuals with ASD, given the highly heterogeneous ASD diagnoses and the lifelong pervasiveness of ASD and SIB. SIB presentation can be extremely variable, including in its duration, and thus the current study is limited in size and scale (though more extensive than comparable existing studies). SIB data here may not have been sufficient for some participants, such as P8, when SIB episodes are few and/or short (< 100 s). There would thus be value in monitoring SIB across several days (longitudinal recordings) to capture additional episodes across different contexts, as well as expanding the study with a larger sample. Data from other episodes may also help increase explanatory power for the MLR, though accuracy is perhaps more critical for online detection of SIB. Although recall, sensitivity, and accuracy remained relatively high when testing MLR with unbalanced, natural data, precision decreased. This decrease in precision indicates that a “quasi-balancing” may be required when implementing classifiers in a real-world settings. At present, this technology would be most useful in settings where individuals frequently exhibit SIB (e.g., school), as there would be a greater need for support in these settings and a more balanced SIB:non-SIB ratio. A classifier could be deployed when caregivers cannot maintain both tracking and behavior management due to the high frequency and/or intensity of behaviors. It may be possible to improve MLR by weighting terms based on the frequency of the behaviors, as well as based on caregiver perception of imminent danger. Other methods of improvement could include nonlinear terms with variable slopes, though this could decrease interpretability of the model. Additional levels could also be incorporated into the model, such as age, SIB type, frequency or intensity. The definition of frequency or the rate of SIB can vary, depending on the type of behavior and the observer (e.g., counting each hit or counting each set of hits); thus, frequency was not included to describe behaviors. Behavior was classified by the presence/absence of SIB in the time window versus using a defined frequency. Similarly, intensity was not quantified objectively, though doing so would be a valuable contribution for future iterations of sensing technology for ASD. Operational definitions of the frequency of SIB and associated intensity, though, would be necessary to establish ground truth for inter-rater reliability. Further, additional analysis of effects of observation time and duration on classifier performance could support decisions about data requirements for training and testing data.

Our work supports the presence of nonlinear motor variability within SIB. However, several features of nonlinear motor variability (DFA) loaded only modestly (< ~ 0.1) on PCs, which may be due to the young ages of the participant pool. The long-range correlations quantified by DFA only develop in gait during late childhood^86^, so such correlations may not yet be evident in SIB movements when the participants are young. Age could be incorporated as a covariate in future work, which may show differences in feature importance, such as in DFA, among age groups. Older participants could show more explicit anti/persistence in pathological movement, which could lead to additional evidence of nonlinear motor variability in SIB. Dynamic movement signatures of individuals with ASD could provide information to detect pathology, such as movements involved in SIB, before typical diagnostic measures^85^, and could explain the pattern of SIB onset. These individual movement signatures might also reveal trends about intentions that underlie SIB movements, such as whether the motion is goal-directed or spontaneous^85^, or the etiology of ASD, through mapping movement characteristics to underlying mechanisms of movement^87^. With additional information about ASD movement signatures, variability components (quantified with metrics such as RQA and entropy) could be the basis for an intervention to promote self-awareness and intentional movements in ASD^88^. Specifically, if SIB is a deterministic process, nonlinear motor variability metrics could capture the convergence or divergence of the repeated motions that may indicate the onset of SIB from sensor data. If values from such metrics surpass a certain threshold, the child would be considered at risk for starting an SIB. Stimuli (e.g., visual or auditory signals) could provide feedback to alert the child and divert the child’s attention to alternative coping mechanisms (e.g., a breathing app, squeezing a sensory object, or feeling a certain texture).

In future work, we plan to use the current findings to build more sophisticated hierarchical models. One useful addition might be including Bayesian priors for individual-specific information. If such models improve accuracy while retaining interpretability, they could be used to determine the necessity of intervention at the earliest indicator of an event. Specifically, the predicted probability score from logistic regression can serve as a criterion for caregiver interjection, by setting a pre-determined threshold (e.g., caregivers should interject if the probability of SIB > 0.8). A monitoring system could also include real-time estimation of variable parameters (intercept and slope) for each individual with ASD and SIB. Parameter coefficients can be estimated by the empirical Bayes approach, which allows the mean value of the prior distribution to equal the mean of coefficients from the training data. Using new data in real-time, the posterior distribution can then be recomputed and updated for that participant. Continuously adapting features could both improve current models and address evolving behavior when tracking SIB. Alternatively, autoregressive models could be employed to account for the temporal dynamics of SIB. As in Rad et al.^28^, such an approach could address the common challenge of class imbalances in detecting SIB. However, applying more advanced models, such as an autoregressive model, would likely detract from the interpretability of the SIB prediction, and such a tradeoff would need to be considered carefully when developing a monitoring system.

## Conclusions

This work provides a framework for, and initial results obtained from, interpretable SIB classification at the group-level, particularly through introducing new features with variable slopes and intercepts in a multi-level classifier. A new application of nonlinear metrics to movement in SIB was employed, specifically to develop a group-level classification model. We found that both linear and nonlinear measures of motor variability and time/frequency-domain features, paired with feature selection and dimensional reduction, explained > 65% of the variance found in SIB movement data, and classified diverse SIBs among a group of 10 participants with ~ 75% accuracy. Our results are promising in terms of the feasibility of developing a continuous monitoring system for SIB that can be applied to different types of behaviors and a range of individuals. This work serves as a proof of concept for the utility of technology to track SIB in ASD, which is necessary to apply this work to future Phase 1 prevention efforts. Future work should continue to build on these results, with added consideration of prior distributions for adaptive modeling.

## Supplementary information

Supplementary file1

## Data Availability

The datasets analyzed for this study are not publically available because they contain sensitive information with identifiable behaviors from minors. Requests to access these datasets should be directed to DS.

## Abbreviations

ASD: Autism spectrum disorder
SIB: Self-injurious behavior
SMM: Stereotypical motor movements
SaEn: Sample entropy
RQA: Recurrence quantification analysis
DFA: Detrended fluctuation analysis
PCA: Principal component analysis
MLR: Multi-level logistic regression
LR-variable intercept: Logistic regression with variable intercept only
LR-no variable terms: Logistic regression without variable slopes or intercepts
LR-stepwise: Stepwise logistic regression
LR-ind: Participant-level logistic regression
SVM: Support vector machine
DT: Decision tree
kNN: K-nearest neighbor
