# Supplementary material for: Multi-level modeling with nonlinear movement metrics to classify self-injurious behaviors in autism spectrum disorder
Source: Sci Rep. 2020 Oct 7;10:16699. doi: 10.1038/s41598-020-73155-4 (PMC7542156; doi:10.1038/s41598-020-73155-4)
Supplement: Supplementary file 1 — Supplementary file1 [file 41598_2020_73155_MOESM1_ESM.docx]

## Supplementary Material

**Multi-Level Modeling with Nonlinear Movement Metrics to Classify Self-Injurious Behaviors in Autism Spectrum Disorder**

Kristine D. Cantin-Garside^1^, PhD, Divya Srinivasan^1^, PhD, Shyam Ranganathan^2^, PhD, Susan W. White^3^, PhD, Maury A. Nussbaum^1^, PhD

^1^Department of Industrial and Systems Engineering, Virginia Tech, Blacksburg, VA, USA

^2^Department of Statistics, Virginia Tech, Blacksburg, VA, USA

^3^Center for Youth Development and Intervention; Department of Psychology, University of Alabama, Tuscaloosa, AL, USA

Technical Appendix: Materials and methods

Self-injurious behavior (SIB) definition

Though definitions of SIB vary^[1,2]^, we selected a definition that reflects prior work^[1,3,4]^. For this study, the definition of SIB includes actions that have potential to – though not specifically intending to – cause harm or pain to the self. SIB may serve different functions, excluding accidental incidents (e.g., tripping and falling) and cultural or aesthetic actions (e.g., ear piercings), and may or may not be performed with the intention to cause harm. SIB types may include classic behaviors (e.g., head banging, self-hitting or biting), fine behaviors (e.g., picking or pinching), and potentially injurious body contact with objects or the environment (e.g., repeatedly hitting into walls). In the current study, an SIB “episode” was defined as a continuous time period of a specific SIB type or of co-occurring SIBs that may consist of potentially repetitive and rhythmic actions.

Study preparation

Caregivers were asked during pre-screening to describe the details of their child’s SIB, including known triggers and preferred materials for sensor placements. We also requested that children wear clothing with pockets for sensor placement. We asked that caregivers bring the following items: toys for free play, items associated with triggering and managing SIB, and preferred clothes or accessories.

Instrumentation details

P1 wore two different sensor configurations (1a and 1b) in one session. P1 rejected pocket and ankle sensors during initial data collection but accepted them before lower body SIB was exhibited in the latter half of the session. P8 rejected all sensors except wrist and pocket sensors, and P8 and P9 were siblings (each wore half of the sensors in consecutive sessions). Added materials (cotton, fleece, or sequins) were attached to the sensors as the participant desired; sensor type, location, and attachment methods addressed caregiver needs determined in our previous study^[5]^, including flexible placement in discrete locations, removable, durable, and transferable across environments.

Study overview

An advanced clinical psychology doctoral student then used well-established tools (ADOS and WASI, or Leite-R if the child was nonverbal) to confirm ASD diagnosis^[6,7]^. If SIB did not occur during the session and caregivers decided to proceed with the Standardized Observation Analogue Procedure (SOAP), caregivers chose everyday demands from a list of triggers (e.g., “Point to the toy”), or offered individual-specific demands that may elicit resistance and relate to SIB (e.g., “Wave to the researcher”). The clinician supervised SOAP to ensure prompt intervention when needed. Further details are given in Johnson et al. (2009) and Cantin-Garside et al. (2020)^[8,9]^.

Supplementary Table 1. Means and standard deviations (SD) for select nonlinear variables using wrist accelerometer data from each participant. Variable names presented as “Variable_[axis]_”. DFA = detrended fluctuation analysis exponent, SaEn = sample entropy, crossSaEn = cross sample entropy, Rec = recurrence, Dtrm = determinism, LdiagMAX = maximum diagonal length, MeanVertTT = trapping time, Div = divergence, Lmnrty = laminarity.

|  |  | **DFA_X_** | **DFA_Y_** | **DFA_Z_** | **SaEn_X_** | **SaEn_Y_** | **SaEn_Z_** | **crossSaEn_XY_** | **crossSaEn_XZ_** | **crossSaen_YZ_** | **Rec_X_** | **Dtrm_X_** |
| --- | --- | --- | --- | --- | --- | --- | --- | --- | --- | --- | --- | --- |
| Non-SIB | Mean | 0.488 | 0.488 | 0.497 | 0.636 | 0.610 | 0.589 | 0.155 | 0.147 | 0.187 | 1.052 | 15.939 |
|  | SD | 0.125 | 0.129 | 0.133 | 0.481 | 0.484 | 0.480 | 0.393 | 0.362 | 0.435 | 0.446 | 20.157 |
| SIB | Mean | 0.483 | 0.478 | 0.488 | 0.622 | 0.653 | 0.585 | 0.140 | 0.170 | 0.169 | 1.056 | 15.836 |
|  | SD | 0.137 | 0.148 | 0.134 | 0.491 | 0.531 | 0.491 | 0.387 | 0.384 | 0.412 | 0.444 | 21.127 |

|  |  | **Ldiag**  **MAX_X_** | **MeanVertTT_X_** | **Rec_Y_** | **LdiagMAX_Y_** | **Div_Y_** | **MeanVertTT_Y_** | **Rec_Z_** | **Dtrm_Z_** | **LdiagMAX_Z_** | **Div_Z_** | **Lmnrty_Z_** | **MeanVertTT_Z_** |
| --- | --- | --- | --- | --- | --- | --- | --- | --- | --- | --- | --- | --- | --- |
| Non-SIB | Mean | 2.942 | 1.316 | 1.068 | 2.927 | 0.117 | 1.345 | 1.080 | 17.653 | 3.328 | 0.119 | 5.926 | 1.390 |
|  | SD | 4.036 | 1.597 | 0.453 | 4.201 | 0.133 | 1.587 | 0.440 | 21.372 | 4.557 | 0.131 | 9.770 | 1.609 |
| SIB | Mean | 3.050 | 1.269 | 1.038 | 2.751 | 0.100 | 1.173 | 1.072 | 17.626 | 3.177 | 0.115 | 5.425 | 1.271 |
|  | SD | 4.620 | 1.580 | 0.457 | 4.232 | 0.129 | 1.554 | 0.441 | 22.085 | 4.405 | 0.131 | 9.494 | 1.576 |

Supplementary Table 2. Loadings for the first 12 principal components (PC) extracted. Note that a scree plot was examined to determine the optimal number of components when considering explained variance^[10]^. Though the reliability of scree tests can be unstable, guidelines suggest it is more accurate and less variable than other component-selection methods^[10]^. Further, the number of included variables does not affect scree test accuracy, and results are interpretable compared to other component selection rules^[10]^. With a large number of variables in our study, and given the exploratory nature of the included features, scree tests were used as a first effort toward selecting components to explain the variability in an SIB movement dataset.

|  |  | **Principal Components** | | | | | | | | | | | |
| --- | --- | --- | --- | --- | --- | --- | --- | --- | --- | --- | --- | --- | --- |
| **Type** | **Feature** | **PC1** | **PC2** | **PC3** | **PC4** | **PC5** | **PC6** | **PC7** | **PC8** | **PC9** | **PC10** | **PC11** | **PC12** |
| **Time Domain** | **Correlation Coefficient XY** | 0.023 | 0.012 | -0.037 | 0.039 | -0.131 | -0.016 | 0.058 | 0.113 | 0.046 | 0.133 | 0.139 | -0.232 |
|  | **Correlation Coefficient XZ** | 0.013 | 0.003 | -0.037 | 0.117 | -0.107 | 0.040 | -0.181 | -0.051 | -0.052 | 0.184 | -0.051 | -0.068 |
|  | **Correlation Coefficient YZ** | 0.002 | -0.076 | 0.041 | -0.066 | -0.045 | -0.082 | 0.086 | -0.004 | -0.002 | -0.022 | -0.049 | 0.635 |
|  | **Local Minima Count X** | -0.135 | 0.137 | 0.139 | 0.122 | -0.109 | 0.186 | 0.022 | 0.002 | 0.060 | -0.030 | -0.183 | 0.057 |
|  | **Mean Absolute Value of X** | 0.040 | -0.137 | 0.351 | -0.193 | -0.131 | -0.031 | 0.109 | -0.014 | 0.058 | 0.158 | -0.256 | -0.040 |
|  | **Peak X** | 0.209 | 0.017 | 0.237 | -0.061 | -0.059 | -0.016 | 0.061 | 0.050 | 0.032 | 0.057 | -0.089 | -0.037 |
|  | **Minimum X** | -0.117 | -0.121 | 0.307 | -0.167 | -0.117 | 0.021 | 0.073 | -0.013 | 0.073 | 0.176 | -0.206 | 0.013 |
|  | **10% X** | -0.115 | -0.139 | 0.216 | -0.287 | -0.098 | 0.018 | -0.084 | 0.108 | -0.016 | -0.180 | 0.321 | -0.041 |
|  | **25% X** | -0.071 | -0.127 | 0.249 | -0.302 | -0.103 | 0.013 | -0.082 | 0.121 | -0.020 | -0.197 | 0.344 | -0.044 |
|  | **99% X** | 0.161 | -0.008 | 0.260 | -0.199 | -0.067 | -0.009 | -0.039 | 0.135 | -0.014 | -0.145 | 0.284 | -0.033 |
|  | **Jerk X** | 0.228 | 0.139 | 0.135 | 0.046 | -0.010 | 0.040 | 0.006 | 0.049 | 0.007 | 0.019 | 0.005 | 0.009 |
|  | **Variance X** | 0.181 | 0.159 | 0.144 | 0.020 | 0.009 | 0.040 | -0.034 | 0.013 | 0.057 | 0.051 | 0.133 | 0.170 |
|  | **Local Minima Count Y** | -0.135 | 0.143 | 0.153 | 0.097 | -0.020 | 0.165 | 0.059 | 0.112 | 0.045 | -0.076 | -0.118 | 0.122 |
|  | **Zero Crossings Y** | 0.121 | 0.113 | 0.048 | -0.119 | -0.124 | -0.014 | 0.102 | -0.039 | -0.064 | 0.076 | -0.227 | -0.160 |
|  | **Mean Absolute Value of Y** | 0.054 | -0.054 | 0.015 | 0.414 | 0.192 | 0.015 | -0.113 | 0.061 | 0.029 | -0.111 | 0.314 | 0.143 |
|  | **1% Y** | -0.147 | -0.004 | 0.061 | -0.186 | 0.355 | 0.083 | -0.280 | -0.102 | -0.112 | 0.038 | -0.102 | -0.019 |
|  | **50% Y** | -0.009 | 0.063 | 0.118 | -0.186 | 0.432 | 0.096 | -0.343 | -0.147 | -0.132 | 0.021 | -0.130 | -0.042 |
|  | **99% Y** | 0.153 | 0.119 | 0.145 | -0.103 | 0.321 | 0.081 | -0.274 | -0.117 | -0.078 | -0.002 | -0.063 | -0.001 |
|  | **Jerk Y** | 0.222 | 0.149 | 0.118 | 0.093 | -0.001 | 0.058 | -0.005 | 0.005 | 0.018 | -0.020 | 0.010 | 0.002 |
|  | **Variance Z** | 0.184 | 0.170 | 0.149 | 0.024 | -0.001 | 0.072 | 0.023 | 0.019 | 0.050 | 0.044 | 0.074 | 0.159 |
|  | **Zero Crossings Z** | 0.138 | -0.051 | 0.147 | 0.208 | 0.110 | -0.071 | 0.027 | 0.000 | 0.031 | 0.064 | -0.033 | -0.049 |
|  | **Mean Absolute Value of Z** | 0.029 | 0.314 | -0.249 | -0.254 | -0.116 | 0.061 | 0.014 | -0.011 | -0.040 | -0.009 | 0.051 | 0.026 |
|  | **RMS Z** | 0.067 | 0.322 | -0.223 | -0.236 | -0.110 | 0.064 | 0.017 | -0.012 | -0.036 | -0.012 | 0.051 | 0.036 |
|  | **Peak Z** | 0.200 | 0.238 | -0.031 | -0.073 | -0.072 | 0.073 | -0.002 | -0.008 | -0.011 | -0.038 | 0.020 | 0.037 |
|  | **Minimum Z** | -0.126 | 0.249 | -0.212 | -0.225 | -0.056 | 0.065 | 0.009 | 0.029 | -0.005 | 0.061 | 0.090 | 0.040 |
|  | **50% Z** | -0.042 | 0.052 | -0.037 | -0.170 | 0.392 | -0.094 | 0.395 | 0.105 | 0.260 | -0.060 | -0.018 | -0.042 |
|  | **90% Z** | 0.032 | 0.056 | -0.017 | -0.154 | 0.392 | -0.099 | 0.415 | 0.087 | 0.254 | -0.069 | -0.029 | -0.038 |
|  | **Jerk Z** | 0.218 | 0.167 | 0.116 | 0.068 | -0.036 | 0.063 | -0.004 | 0.010 | 0.011 | -0.017 | -0.018 | -0.004 |
| **Frequency Domain** | **First FFT Amplitude X** | 0.216 | 0.106 | 0.056 | 0.042 | 0.035 | -0.039 | 0.043 | 0.019 | -0.010 | 0.030 | 0.030 | 0.011 |
|  | **Second FFT Amplitude X** | 0.234 | 0.110 | 0.095 | 0.029 | 0.024 | -0.015 | 0.019 | 0.041 | 0.009 | 0.051 | 0.069 | 0.018 |
|  | **Second FFT Peak X** | -0.049 | 0.032 | 0.111 | 0.049 | -0.100 | 0.091 | -0.026 | 0.080 | 0.013 | -0.136 | -0.221 | -0.022 |
|  | **First FFT Amplitude Y** | 0.211 | 0.076 | 0.044 | 0.000 | -0.011 | -0.053 | -0.030 | -0.065 | 0.011 | 0.044 | 0.034 | 0.032 |
|  | **First FFT Peak Y** | -0.098 | 0.079 | 0.078 | 0.189 | 0.076 | 0.181 | -0.023 | 0.153 | 0.101 | -0.186 | 0.046 | -0.124 |
|  | **Second FFT Peak Y** | -0.054 | 0.061 | 0.059 | 0.168 | -0.007 | 0.179 | -0.023 | 0.134 | 0.057 | -0.261 | -0.078 | -0.118 |
|  | **First FFT Peak Z** | -0.078 | 0.188 | -0.036 | -0.011 | -0.007 | 0.184 | -0.075 | 0.148 | 0.101 | -0.130 | -0.052 | -0.186 |
|  | **Second FFT Peak Z** | -0.045 | 0.125 | 0.011 | 0.023 | -0.052 | 0.131 | -0.018 | 0.131 | 0.040 | -0.293 | -0.171 | -0.164 |
| **Nonlinear Variability** | **DFA Alpha X** | 0.183 | -0.041 | -0.107 | -0.014 | 0.003 | -0.126 | 0.025 | -0.049 | -0.096 | -0.156 | -0.051 | -0.041 |
|  | **DFA Alpha Y** | 0.195 | -0.045 | -0.080 | -0.032 | -0.068 | -0.098 | -0.026 | -0.115 | -0.048 | -0.105 | -0.069 | -0.053 |
|  | **DFA Alpha Z** | 0.175 | -0.128 | -0.009 | 0.026 | -0.018 | -0.095 | 0.046 | -0.116 | -0.069 | -0.170 | -0.112 | -0.019 |
|  | **Sample Entropy X** | -0.096 | 0.079 | 0.095 | 0.056 | 0.020 | 0.037 | 0.022 | -0.017 | 0.104 | 0.369 | 0.123 | -0.105 |
|  | **Sample Entropy Y** | -0.098 | 0.079 | 0.100 | 0.091 | 0.070 | 0.044 | 0.058 | 0.041 | 0.044 | 0.317 | 0.124 | -0.020 |
|  | **Sample Entropy Z** | -0.089 | 0.167 | 0.019 | 0.036 | 0.027 | 0.018 | -0.023 | 0.055 | 0.061 | 0.369 | 0.178 | -0.084 |
|  | **Cross Sample Entropy XY** | 0.111 | 0.068 | -0.067 | -0.032 | -0.011 | -0.075 | -0.049 | -0.100 | -0.058 | -0.027 | -0.085 | -0.011 |
|  | **Cross Sample Entropy XZ** | 0.112 | -0.031 | -0.009 | 0.079 | 0.125 | -0.085 | 0.106 | -0.005 | 0.012 | -0.107 | 0.042 | -0.064 |
|  | **Cross Sample Entropy YZ** | 0.099 | -0.043 | 0.047 | 0.000 | -0.072 | -0.026 | 0.049 | -0.093 | -0.001 | -0.020 | -0.084 | -0.326 |
|  | **Recurrence X** | 0.049 | -0.069 | -0.077 | -0.030 | 0.011 | 0.014 | -0.112 | 0.495 | -0.071 | 0.147 | -0.189 | 0.130 |
|  | **Determinism X** | 0.160 | -0.132 | -0.136 | -0.054 | 0.068 | -0.037 | -0.095 | 0.333 | -0.049 | 0.096 | 0.009 | -0.071 |
|  | **Maximum Diagonal Length X** | 0.147 | -0.125 | -0.123 | -0.052 | 0.060 | -0.026 | -0.100 | 0.386 | -0.069 | 0.119 | -0.022 | -0.057 |
|  | **Trapping Time X** | 0.088 | -0.098 | -0.094 | -0.060 | 0.034 | 0.043 | -0.082 | 0.374 | -0.044 | 0.024 | -0.202 | 0.156 |
|  | **Recurrence Y** | 0.044 | -0.067 | -0.053 | -0.035 | -0.056 | 0.061 | -0.233 | -0.051 | 0.581 | 0.000 | -0.044 | 0.075 |
|  | **Maximum Diagonal Length Y** | 0.146 | -0.113 | -0.101 | -0.063 | -0.045 | -0.017 | -0.166 | -0.031 | 0.293 | 0.058 | 0.023 | -0.065 |
|  | **Divergence Y** | 0.083 | -0.098 | -0.120 | -0.066 | -0.043 | -0.034 | -0.151 | -0.094 | 0.257 | 0.010 | 0.000 | -0.091 |
|  | **Trapping Time Y** | 0.090 | -0.098 | -0.095 | -0.054 | -0.059 | 0.058 | -0.190 | -0.084 | 0.461 | -0.035 | -0.043 | 0.079 |
|  | **Recurrence Z** | 0.041 | -0.120 | -0.043 | -0.027 | 0.022 | 0.429 | 0.140 | -0.072 | -0.031 | 0.078 | 0.048 | -0.045 |
|  | **Determinism Z** | 0.157 | -0.213 | -0.080 | -0.013 | 0.034 | 0.191 | 0.084 | -0.013 | -0.062 | 0.053 | 0.055 | -0.138 |
|  | **Maximum Diagonal Length Z** | 0.147 | -0.185 | -0.079 | -0.010 | 0.031 | 0.229 | 0.091 | -0.004 | -0.065 | 0.078 | 0.050 | -0.189 |
|  | **Divergence Z** | 0.079 | -0.154 | -0.036 | -0.034 | 0.023 | 0.116 | 0.092 | -0.151 | -0.085 | -0.024 | 0.071 | 0.053 |
|  | **Laminarity Z** | 0.099 | -0.129 | -0.066 | -0.046 | 0.014 | 0.421 | 0.119 | -0.041 | -0.011 | 0.013 | -0.004 | 0.143 |
|  | **Trapping Time Z** | 0.081 | -0.151 | -0.073 | -0.050 | 0.013 | 0.436 | 0.138 | -0.106 | -0.044 | 0.032 | 0.036 | 0.142 |
| **Explained Variance (%)** | | **23.011** | **8.014** | **6.323** | **5.270** | **3.900** | **3.562** | **3.249** | **3.006** | **2.624** | **2.455** | **2.269** | **1.876** |

**Supplementary Table 3**. Descriptive statistics for head banging versus non-SIB events for P9. DFA = detrended fluctuation analysis exponent, SaEn= sample entropy, crossSaEn = cross sample entropy, RecRate = recurrence rate (%), Dtrm = determinism (%), LdiagMAX = maximum diagonal length, Div = divergence (%), Lmnrty = laminarity (%), MeanVertTT = trapping time. Variables are formatted as “VariableName_[axis]_[participant number][sensor number]”. Sensor number 3 = waist.

|  |  | **DFA_X_9_3** | **DFA_Y_9_3** | **DFA_Z_9_3** | **SaEn_X_9_3** | **SaEn_Y_9_3** | **SaEn_Z_9_3** | **crossSaEn_XZ_9_3** | **crossSaEn_YZ_9_3** |
| --- | --- | --- | --- | --- | --- | --- | --- | --- | --- |
| **Non-SIB** | **Mean** | 0.656 | 0.372 | 0.350 | 0.393 | 0.843 | 0.891 | 0.006 | 0.060 |
|  | **SD** | 1.332 | 0.085 | 0.080 | 0.101 | 0.514 | 0.505 | 0.078 | 0.254 |
|  | **Min.** | 0.070 | 0.000 | 0.000 | 0.000 | -0.062 | -0.061 | 0.000 | -0.072 |
|  | **Max.** | 21.836 | 0.805 | 0.872 | 0.848 | 3.080 | 3.235 | 1.774 | 2.793 |
| **SIB** | **Mean** | 0.457 | 0.441 | 0.482 | 0.567 | 0.612 | 0.581 | 0.038 | 0.203 |
|  | **SD** | 0.127 | 0.072 | 0.116 | 0.467 | 0.459 | 0.469 | 0.156 | 0.399 |
|  | **Min.** | 0.280 | 0.308 | 0.322 | 0.000 | 0.000 | 0.150 | 0.000 | 0.000 |
|  | **Max.** | 0.805 | 0.527 | 0.728 | 1.483 | 1.512 | 1.848 | 0.643 | 1.390 |

|  |  | **RecRate_X_9_3** | **Dtrm_X_9_3** | **LdiagMAX_X_9_3** | **Div_X_9_3** | **Lmnrty_X_9_3** | **MeanVertTT_X_9_3** |
| --- | --- | --- | --- | --- | --- | --- | --- |
| **Non-SIB** | **Mean** | 2.234 | 1.049 | 6.890 | 1.275 | 0.121 | 3.066 |
|  | **SD** | 2.542 | 0.463 | 14.438 | 2.770 | 0.356 | 6.862 |
|  | **Min.** | 1.000 | 0.000 | 0.000 | 0.000 | 0.000 | 0.000 |
|  | **Max.** | 42.771 | 1.905 | 77.273 | 28.000 | 2.322 | 60.976 |
| **SIB** | **Mean** | 1.011 | 3.587 | 0.765 | 0.074 | 1.740 | 0.706 |
|  | **SD** | 0.564 | 7.805 | 1.437 | 0.138 | 3.255 | 1.312 |
|  | **Min.** | 0.256 | 0.000 | 0.000 | 0.000 | 0.000 | 0.000 |
|  | **Max.** | 1.868 | 27.500 | 4.000 | 0.333 | 8.333 | 3.000 |

|  |  | **RecRate_Y_9_3** | **Dtrm_Y_9_3** | **LdiagMAX_Y_9_3** | **Div_Y_9_3** | **Lmnrty_Y_9_3** | **MeanVertTT_Y_9_3** |
| --- | --- | --- | --- | --- | --- | --- | --- |
| **Non-SIB** | **Mean** | 4.459 | 1.039 | 3.748 | 0.744 | 0.062 | 2.073 |
|  | **SD** | 7.146 | 0.470 | 10.455 | 2.098 | 0.258 | 5.539 |
|  | **Min.** | 1.019 | 0.037 | 0.000 | 0.000 | 0.000 | 0.000 |
|  | **Max.** | 70.714 | 1.905 | 76.471 | 25.000 | 2.000 | 45.455 |
| **SIB** | **Mean** | 0.995 | 9.822 | 2.118 | 0.056 | 2.435 | 1.078 |
|  | **SD** | 0.596 | 18.434 | 4.256 | 0.104 | 3.855 | 1.507 |
|  | **Min.** | 0.220 | 0.000 | 0.000 | 0.000 | 0.000 | 0.000 |
|  | **Max.** | 1.868 | 59.459 | 16.000 | 0.333 | 11.905 | 3.333 |

|  |  | **RecRate_Z_9_3** | **Dtrm_Z_9_3** | **LdiagMAX_Z_9_3** | **Div_Z_9_3** | **Lmnrty_Z_9_3** | **MeanVertTT_Z_9_3** |
| --- | --- | --- | --- | --- | --- | --- | --- |
| **Non-SIB** | **Mean** | 2.030 | 1.049 | 7.351 | 1.376 | 0.131 | 3.215 |
|  | **SD** | 1.516 | 0.463 | 15.445 | 3.054 | 0.374 | 7.183 |
|  | **Min.** | 1.000 | 0.000 | 0.000 | 0.000 | 0.000 | 0.000 |
|  | **Max.** | 17.133 | 1.905 | 86.842 | 29.000 | 2.000 | 62.903 |
| **SIB** | **Mean** | 0.907 | 16.283 | 3.118 | 0.191 | 1.875 | 0.424 |
|  | **SD** | 0.387 | 16.019 | 2.913 | 0.147 | 6.339 | 1.204 |
|  | **Min.** | 0.256 | 0.000 | 0.000 | 0.000 | 0.000 | 0.000 |
|  | **Max.** | 1.575 | 60.606 | 10.000 | 0.333 | 25.806 | 4.000 |

**References**

1 Rojahn, J., Matson, J. L., Lott, D., Esbensen, A. J. & Smalls, Y. The Behavior Problems Inventory: An instrument for the assessment of self-injury, stereotyped behavior, and aggression/destruction in individuals with developmental disabilities. *Journal of Autism and Developmental Disorders* **31**, 577-588 (2001).

2 Lam, K. S. & Aman, M. G. The Repetitive Behavior Scale-Revised: Independent validation in individuals with autism spectrum disorders. *Journal of Autism and Developmental Disorders* **37**, 855-866 (2007).

3 Iwata, B. A. *et al.* The functions of self-injurious behavior: An experimental-epidemiological analysis. *Journal of Applied Behavior Analysis* **27**, 215-240 (1994).

4 Pace, G. M., Iwata, B. A., Edwards, G. L. & McCosh, K. C. Stimulus fading and transfer in the treatment of self‐restraint and self‐injurious behavior. *Journal of Applied Behavior Analysis* **19**, 381-389 (1986).

5 Cantin-Garside, K., Nussbaum, M., White, S., Kim, S., Kim, C.D., Fortes, D., Valdez, R.S. (In Press). Understanding the experiences of self-injurious behavior in autism spectrum disorder: Implications for monitoring technology design. *Journal of the American Medical Informatics Association* (2020).

6 Lord, C. et al. Autism diagnostic observation schedule–2nd edition (ADOS-2). *Los Angeles, CA: Western Psychological Corporation* (2012).

7 Pugliese, C. E. *et al.* Replication and comparison of the newly proposed ADOS-2, Module 4 algorithm in ASD without ID: A multi-site study. *Journal of Autism and Developmental Disorders* **45**, 3919-3931 (2015).

8 Johnson, C. R. et al. Standardised Observation Analogue Procedure (SOAP) for assessing parent and child behaviours in clinical trials. *Journal of Intellectual and Developmental Disabilities* **34**, 230-238 (2009).

9 Cantin-Garside, K. D., Kong, Z., White, S. W., Antezana, L., Kim, S., & Nussbaum, M. A. Detecting and classifying self-injurious behavior in autism spectrum disorder using machine learning techniques. *Journal of Autism and Developmental Disorders*, 1-14 (2020).

10 Zwick, W. R. & Velicer, W. F. Comparison of five rules for determining the number of components to retain. *Psychological Bulletin* **99**, 432-442 (1986).
